# Marker-assisted breeding of new wheat germplasm with adult-plant resistance to stripe rust in the genetic background of Longmai479

**DOI:** 10.3389/fpls.2026.1954529

**Published:** 2026-09-16

**Authors:** Caihong Chen, Boxun Yu, Peiyao Huang, Yiduo Zhang, Kebing Huang, Suizhuang Yang

**Affiliations:** Wheat Research Institute, College of Life Sciences and Agri-Forestry, Southwest University of Science and Technology, Mianyang, China

**Keywords:** agronomic trait evaluation, disease resistance identification, marker-assisted breeding, wheat stripe rust, *Yr* genes

## Abstract

In response to the worsening severity of wheat stripe rust in recent years, which poses a serious threat to food security, new wheat germplasm with adult-plant resistance and superior agronomic traits is being developed. We developed five hybrid combinations by crossing Longmai479 with 50212-2, Xikemai11, Chuanmai98, Chuanyu34, and 50212-12, yielding 164 families. We subjected mixed seeds from the F_2_ to F_4_ generations to artificial selection, followed by manual screening for superior disease-resistant individual plants in the F_5_ generation. In the F_6_ generation, marker-assisted selection was performed using molecular markers tightly linked to *Yr10*, *Yr15*, *Yr17*, *Yr18*, *Yr26*, *Yr29*, and *Yr30* for PCR amplification and genotyping. By combining field-based disease resistance identification with agronomic trait evaluation, wheat families exhibiting both disease resistance and superior agronomic traits, including those with single-gene or multi-gene pyramiding, were comprehensively selected. Ultimately, 18 families with disease resistance and superior agronomic traits were obtained. Among these, two families carried none of the target *Yr* genes, two carried one *Yr* gene, seven carried two *Yr* genes, four carried three *Yr* genes, and three carried four *Yr* genes. These new wheat germplasm lines, which exhibit disease resistance and superior agronomic traits, hold promise for development into high-quality new cultivars. They will make significant contributions to stripe rust control and wheat production by providing important genetic resources for breeding disease-resistant wheat varieties.

## 1 Introduction

As a staple crop for 40% of the global population, wheat (*Triticum aestivum* L.) plays a critical role in ensuring food security, making yield enhancement highly significant. Wheat stripe rust, caused by *Puccinia striiformis* f. sp. *Tritici* (Pst), is one of the most important fungal diseases affecting wheat production. This disease occurs worldwide, including in major wheat-producing regions such as China, India, the United States, and Australia (Ali et al., 2017). Outbreaks of stripe rust typically result in grain yield losses ranging from 40% to 100% (Li et al., 2018). In Australia, the stripe rust epidemic of 1984–1986 caused wheat yield losses of up to 80% (Wellings and McIntosh, 1990). China is the largest epidemic region for stripe rust globally; since 1950, five major epidemics have occurred, each affecting more than 5.5 million hectares and resulting in a cumulative wheat loss of 13.8 million metric tons (Ma, 2018; Liu et al., 2022).

The most effective approach for controlling wheat stripe rust is the development of resistant cultivars. However, the high variability of Pst leads to the continuous emergence of new physiological races, causing resistance in developed cultivars to break down within three to six years (Kang et al., 2015). In the 1990s, the emergence of races CYR30, CYR31, and CYR32 rendered resistance ineffective in Fan6-derived families and cultivars carrying *Yr9* or *Yr17*, resulting in severe yield losses (Kang et al., 2022; Han and Kang, 2018). Since 2009, CYR34 has become the predominant race, leading to a gradual loss of resistance in cultivars based on resistance genes such as *Yr10* and *Yr26* (Jiang et al., 2024; Liu et al., 2017) Currently, the all-stage resistance (ASR) genes *Yr5*, *Yr10*, *Yr15*, and *Yr26* still confer strong resistance against CYR32 and CYR33, with *Yr5* and *Yr15* also exhibiting ASR against the G22 pathotype (Zeng et al., 2015).

To date, 87 stripe rust resistance genes have been officially named (*Yr1*–*Yr87*), including 60 ASR genes and 27 adult-plant resistance (APR) genes (Sharma et al., 2024). The ASR genes detected in this study were *Yr10*, *Yr15*, *Yr17*, and *Yr26*, while the APR genes were *Yr18*, *Yr29*, and *Yr30*. All markers used in the experiment were validated tightly linked molecular markers: *SC200* is genetically only 0.5 cM from *Yr10* (Shao et al., 2001); the codominant marker *csLv34* is 2.5 cM from *Yr18* (Kolmer et al., 2008); the SSR marker *Xbarc8* is 9.9 cM from *Yr15* (Peng et al., 2000); *SC-385* is the closest linked marker to *Yr17* (Jia et al., 2011); and *WE173* is less than 0.5 cM from *Yr26*, which can significantly improve the efficiency of marker-assisted selection (MAS) for *Yr26* (Han et al., 2015). Furthermore, the SSR marker *WMC44*, which is linked to the multi-disease resistance gene cluster *Lr46/Yr29/Pm39* conferring APR, is 5–15 cM from the target locus (Rosewarne et al., 2006). The APR locus *Yr30*, located on chromosome 3BS, is tightly linked to *Sr2*; the selected marker *WMS533* (Crossa et al., 2007) is 2.0 cM from this locus, enabling stable detection of the target genotype.

MAS enables detection at any growth stage, thereby improving efficiency. Moreover, by enabling direct selection for target genes unaffected by environmental conditions, MAS provides elite parental material for variety development. As an efficient and precise breeding approach, this technique has been widely applied to various crops, including barley, cotton, rice, and wheat (Yasuda et al., 2015; Chemayek et al., 2016; Chen, 2013). Extensive practical applications have validated its value: He et al. (2026) combined conventional breeding with MAS to pyramid genes for high-molecular-weight glutenin subunits and multiple disease-resistance genes, developing the new wheat cultivar “Lantian214” and synergistically improving disease resistance, quality, and yield. Brar et al. (2019) demonstrated that pyramiding *Fhb2*+*Fhb5* and *Fhb2*+*Fhb5*+*Fhb1* genes significantly reduces the severity of Fusarium head blight in wheat and decreases deoxynivalenol (DON) accumulation in diseased kernels. Wang et al. (2023) investigated the results of APR tests conducted in field areas with a high incidence of stripe rust, demonstrating that pyramiding multiple partial resistance genes (*Yr28*+*Yr36* and *Yr18*+*Yr28*+*Yr36*) can enhance wheat stripe rust resistance. Notably, the pyramiding of APR genes can confer ASR, which is beneficial for wheat resistance breeding.

Although the *Yr30* gene confers relatively low resistance as a single gene and exhibits high susceptibility at the seedling stage, pyramiding this gene produces an additive effect that enhances the plant’s disease resistance (Liu et al., 2024). Zhang (2022a) evaluated stripe rust resistance and conducted gene detection in candidate cultivars from major winter wheat-producing regions of China. The study revealed that *Yr7*, *Yr9*, *Yr29*, and *Yr30* are widely distributed in these regions, as they were detected in cultivars from all provinces. Although molecular detection has been progressively adopted in Sichuan-bred wheats, the *Yr30* detection rate in Sichuan cultivars remained lower than in Gansu. In an evaluation of 39 Sichuan wheat cultivars, Xi et al. (2020) observed that *Yr30* was potentially present in half of the accessions, and 20 accessions exhibited APR after pyramiding *Yr30* with other genes. Similarly, in a national evaluation of stripe rust resistance involving 305 wheat cultivars, Zhang Yue tested 40 accessions from the Chuannong series and reported that 17 carried *Yr30* (Zhang, 2022b).

Building on the aforementioned research foundation, this study aims to introduce *Yr30*, which has the wheat cultivar Longmai479 (LM479) as its genetic background, into Sichuan wheat using MAS, thereby creating new germplasm that combines APR with superior agronomic traits. This work provides a theoretical basis and germplasm resources for the breeding of wheat cultivars with durable disease resistance.

## 2 Materials and methods

### 2.1 Plant materials

Five hybrid combinations were developed using LM479 as the female parent and 50212-2, Xikemai11 (XKM11), Chuanmai98 (CM98), Chuanyu34 (CY34), and 150212–12 as the male parents (**Table 1**). Genotyping for the target stripe rust resistance genes *Yr10*, *Yr15*, *Yr17*, *Yr18*, *Yr26*, *Yr29*, and *Yr30* was conducted using seven previously reported functional markers or tightly linked SSR markers. Field phenotyping for disease resistance was performed at the APR. By integrating genotypic and phenotypic data, the distribution and pyramiding status of resistance genes in the tested materials were determined. The susceptible control used was Jinmai47 (JM47), provided by the Wheat Research Institute of Southwest University of Science and Technology.

**Table 1:** Parental lines used in this study and their characteristics.

Parental line	Source	Pedigree	Key characteristics	*Yr* gene
LM479	Pingliang Academy of Agricultural Sciences	Changwu134/TW98-829-1	High and stable yield, resistance to stripe rust	*Yr30* (Wang et al., 2021; Bai et al., 2024)
XKM11	Institute of Wheat, Southwest University of Science and Technology	Guinong06/J20	Highly resistant to stripe rust and powdery mildew	*Yr10*, *Yr17*, *Yr26*, *Yr29* (Zhang et al., 2022; Yang et al., 2024)
CM98	Research Institute of Sichuan Academy of Agricultural Sciences	Jian3/Chuannong19//Chuanmai104	Highly resistant to stripe rust and powdery mildew	*Yr10*, *Yr17*, *Yr26*, *Yr29* (Zhang et al., 2022; Yang et al., 2024; Zhou et al., 2020)
CY34	Chengdu Institute of Biology, Chinese Academy of Sciences	—	Highly resistant to stripe rust	*Yr15*, *Yr1*8 (Zhang et al., 2022)
50212-2	Institute of Wheat, Southwest University of Science and Technology	—	Disease-resistant germplasm	Unknown
50212-12	Institute of Wheat, Southwest University of Science and Technology	—	Disease-resistant germplasm	Unknown

LM479, Longmai479; CM98, Chuanmai98; XKM11, Xikemai11; CY34, Chuanyu34.

### 2.2 Population construction and field planting

This experiment was conducted from 2020 to 2026 at the wheat breeding base of Southwest University of Science and Technology, located in Jiangyou City, Mianyang, Sichuan Province (31.41°N, 104.39°E). The terrain is flat, with warm and humid winters, abundant rainfall, and high field humidity. As an overwintering area for Pst, the disease can occur naturally under local conditions. To promote uniform and sufficient occurrence of stripe rust throughout the experimental area, one row of the susceptible cultivar JM47 was evenly sown in each aisle as a trap row, and artificial inoculation was performed in early January each year. Inoculation was conducted in the evening (17:00–18:00) to take advantage of the low temperature and high humidity at night, which facilitate spore germination. Fresh urediniospores of Pst. tritici physiological races CYR32, CYR33, and CYR34 (provided by the Institute of Plant Protection, Gansu Academy of Agricultural Sciences) were mixed in equal proportions, thoroughly blended with talc powder at a 1:100 ratio, and evenly dusted onto the leaves of JM47 using the powder-shaking method. No diseased leaves were collected from the field for isolation and identification of prevalent races during the experimental period.

From heading to flowering in March 2020, LM479 was used as the female parent for emasculation and bagging, with relevant information recorded. When the stigmas of the emasculated flowers became feathery, branched, and glossy, mature pollen from the male parents 50212-2, XKM11, CM98, CY34, and 50212–12 was collected and applied to the stigmas of LM479 (**Figure 1**). The plants were re-bagged, and F_1_ seeds were harvested in mid-May. Bulk harvesting with artificial selection was performed for generations. The bulk method was employed for breeding in the F_2_–F_4_ generations. Individual plant selection was conducted in the F_5_ generation. In the F_6_ generation, the selected F_5_ plants were planted as progeny rows, and superior individuals were further selected for agronomic traits and disease resistance. For the F_2_–F_5_ seeds derived from the five hybrid combinations, each row was sown 2 meters apart, with approximately 80 seeds per row across 30 rows, and a row spacing of 30 cm. During selection, plants exhibiting disease susceptibility and a plant height (PH) >110 cm were discarded. To preserve all possible genotypes, seeds were bulked from each generation.

**Figure 1:**
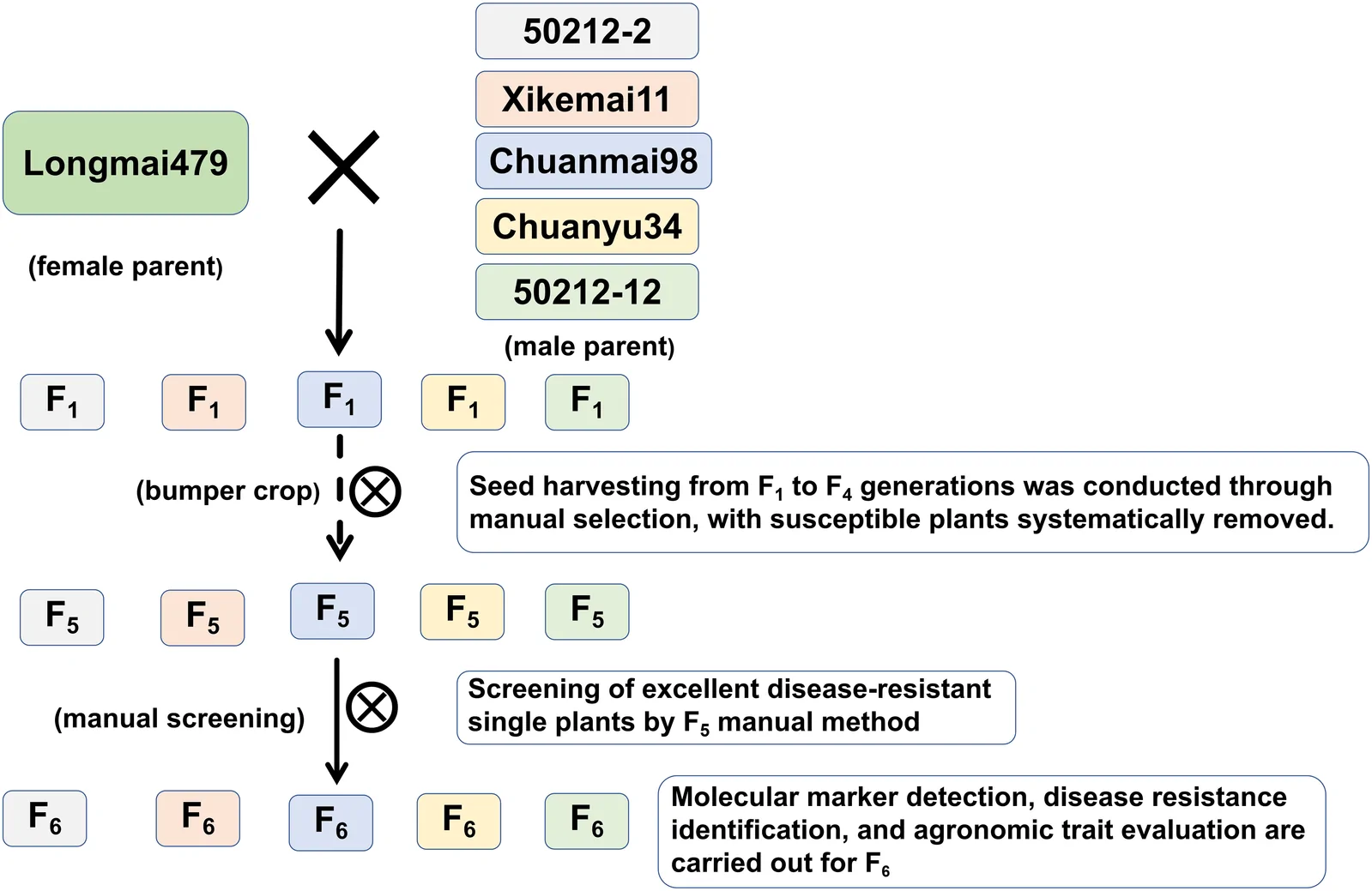
Hybrid breeding pipeline for developing multi-disease resistance gene pyramiding populations based on marker-assisted selection (MAS).

During the 2024–2025 growing season, 164 elite F_5_ individual plants were selected based on the criteria of infection type (IT) ≤ 6, PH ≤ 100 cm, tiller number (NT) ≥ 3, and spike length (SL) ≥ 10 cm, and their seeds were harvested to produce the F_6_ generation. Of these, 27 families were derived from LM479/50212-2, 49 from LM479/XKM11, 32 from LM479/CM98, 24 from LM479/CY34, and 32 from LM479/50212-12.

During the 2025–2026 growing season, the experimental materials were arranged in a randomized block design with three replications. Each plot consisted of three rows, with a row length of 1.2 m and a row spacing of 0.3 m. Thirty seeds were uniformly broadcast per row. The walkway between replications was 0.4 m wide. The total area of the test site is 767.52 m². Field management, including irrigation and fertilization, followed local conventional practices. All experimental materials were planted in the same experimental field, with replications arranged sequentially along the long axis of the field to minimize experimental errors caused by environmental heterogeneity among different fields.

### 2.3 Molecular marker detection

Genomic DNA was extracted from all parental families and F_6_ families using a modified cetyltrimethylammonium bromide (CTAB) method (Mace et al., 2003). After extraction, the concentration and purity of the DNA samples were measured using a visible spectrophotometer. Upon confirming that the sample quality met the requirements for subsequent experiments, the stock DNA solutions were uniformly diluted with sterile double-distilled water (ddH_2_O) to a standardized working concentration of 80 ng·µL^−1^ and stored at -4°C for future use. Simple sequence repeat (SSR), cleaved amplified polymorphic sequence (CAPS), sequence-tagged site (STS), and corresponding functional markers closely linked to the target stripe rust resistance genes *Yr30*, *Yr29*, *Yr26*, *Yr18*, *Yr17*, *Yr15*, and *Yr10* were selected for molecular genotyping of the families. All primers were synthesized by Sangon Biological Engineering (Shanghai) Co, Ltd. (Shanghai, China) (https://www.sangon.com/) (**Table 2**).

**Table 2:** Sequence and amplification information for markers linked to *Yr30, Yr29, Yr26, Yr18, Yr17, Yr15*, and *Yr10*.

Gene	Type	Marker	Primer sequence	(Tm°C)	References
*Yr10*	SCAR	*SC200*	F: CTGCAGAGTGACATCATACA R: TCGAACTAGTAGATGCTGGC	62	(Zhang et al., 2014)
*Yr15*	SSR	*XBarc8*	F: GCGGGAATCATGCATAGGAAAACAGAA R: GCGGGGGCGAAACATACACATAAAAACA	58	(Peng et al., 2000)
*Yr17*	SCAR	*SC-385*	F: CTGAATACAAACAGCAAACCAG R: ACAGAAAGTGATCATTTCCATC	53	(Jia et al., 2011)
*Yr18*	STS	*csLv34*	F: GTTGGTTAAGACTGGTGATGG R: TGCTTGCTATTGCTGAATAGT	57	(Lagudah et al., 2009)
*Yr26*	STS	*Xwe173*	F: GGGACAAGGGGAGTTGAAGC R: GAGAGTTCCAAGCAGAACAC	60	(Han et al., 2015)
*Yr29*	AFRP	*Wmc44*	F: GGTCTTCTGGGCTTTGATCCTG R: TGTTGCTAGGGACCCGTAGTGG	58	(William et al., 2003)
*Yr30*	SSR	*Xgwm533*	F: GTTGCTTTAGGGGAAAAGCC R: AAGGCGAATCAAACGGAATA	57	(Hayden et al., 2004)

The total volume of the polymerase chain reaction (PCR) reaction was 10 µL, consisting of 5 µL of 2× San Taq PCR Mix, 0.8 µL of DNA template, 0.8 µL of primers (0.4 µL each of forward and reverse), and 3.4 µL of ddH_2_O. The PCR protocol included an initial denaturation at 94 °C for 3 min, followed by 35 cycles comprising denaturation at 94 °C for 30 s, annealing at 58–65 °C for 30 s (with the annealing temperature varying depending on the primers), and extension at 72 °C for 30–60 s per kilobase pair. After the cycles, a final extension was performed at 72 °C for 10 min, and the samples were stored at 4 °C pending analysis. The products were analyzed by 1.5% agarose gel electrophoresis. Gel images were captured using the ChemiDoc MP universal gel imaging and analysis system and scanned and archived using Image Lab software (version 5.0 build 18).

### 2.4 Disease resistance evaluation

During the peak incidence period of wheat stripe rust in Mianyang, Sichuan Province, in April 2026, field evaluations of APR were conducted on 164 F_6_ families. Resistance performance was assessed using a 0–9 scale for IT. The investigation of DS in the parents and families commenced when the susceptible control, JM47, exhibited full disease expression (DS ≥ 80%). IT of the parents and each family was recorded on a 0–9 scale (Qayoum and Line, 1985), in which IT = 0 indicated immunity, 1–3 indicated high resistance, 4–6 indicated moderate resistance, and 7–9 indicated susceptibility. DS was rated according to the percentage of leaf area covered by pustules, with scale values of 0, 5, 10, 20, 30, 40, 50, 60, 70, 80, 90, and 100% (Peterson et al., 1948). Both IT and DS were surveyed three times per replicate, and the mean values were used as the final results.

### 2.5 Investigation of agronomic traits

During the wheat maturity period in May 2026, an agronomic trait evaluation was conducted on 164 F_6_ families from five hybrid combinations at the Mianyang trial site in Sichuan Province. PH, SL, spikelet number (SN), and NT were measured in the field. For each family, three plants were randomly selected for measurement, and the average values were calculated. After harvesting, threshing, and drying the grains, the grain number per spike (GNPS) was determined using a PME automatic grain counter. Thousand-grain weight (TGW): One hundred wheat grains were randomly weighed using an electronic balance, and the result was multiplied by 10. The measurement was repeated three times, and the average value was recorded as the TGW.

### 2.6 Data analysis

The raw data on disease resistance evaluation and agronomic traits of each line were compiled using Excel 2010. One-way analysis of variance (ANOVA) was employed to evaluate significant differences among multiple treatment groups. The assumptions of normality and homogeneity of variances were assessed using the Shapiro–Wilk test and Bartlett’s test, respectively. The results confirmed that the data satisfied both assumptions (P > 0.05), justifying the use of ANOVA in this study. All measured values are presented as means. Graphs were generated using Origin 2021.

## 3 Results

### 3.1 Molecular marker detection

To clarify the distribution of target stripe rust resistance genes in the progeny families, this study conducted molecular marker detection on 164 F_6_ families from five hybrid combinations using markers *SC200*, *XBarc8*, *SC-385*, *csLv34*, *We173*, *Wmc44*, and *Xgwm533*, which are tightly linked to *Yr10*, *Yr15*, *Yr17*, *Yr18*, *Yr26*, *Yr29*, and *Yr30*, respectively (**Figures 2a–g**). *Yr30* was detected in 72 of the 164 materials, corresponding to a detection rate of 43.9%, while 29 materials contained none of the target *Yr* genes. Detection results for 81 families from the two hybrid combinations LM479/XKM11 and LM479/CM98 indicated that four families contained no *Yr* genes, seven families contained only one *Yr* gene, 32 families carried two *Yr* resistance genes (20 from LM479/XKM11 and 12 from LM479/CM98), 26 families carried three *Yr* resistance genes (15 from LM479/XKM11 and 11 from LM479/CM98), and 12 families carried four *Yr* resistance genes (7 from LM479/XKM11 and 5 from LM479/CM98). Among the 81 families, the detection counts for *Yr10*, *Yr17*, *Yr26*, and *Yr29* were 54, 45, 31, and 36, corresponding to detection rates of 66.7%, 55.6%, 38.3%, and 44.4%, respectively. *Yr10* had the highest detection rate, whereas *Yr26* had the lowest. Among the 24 families derived from the LM479/CY34 cross, two carried no *Yr* genes, seven contained only one of the three *Yr* genes (*Yr15, Yr18*, or *Yr30*), six had two *Yr* genes, and the remaining nine pyramided all three target *Yr* genes.

**Figure 2:**
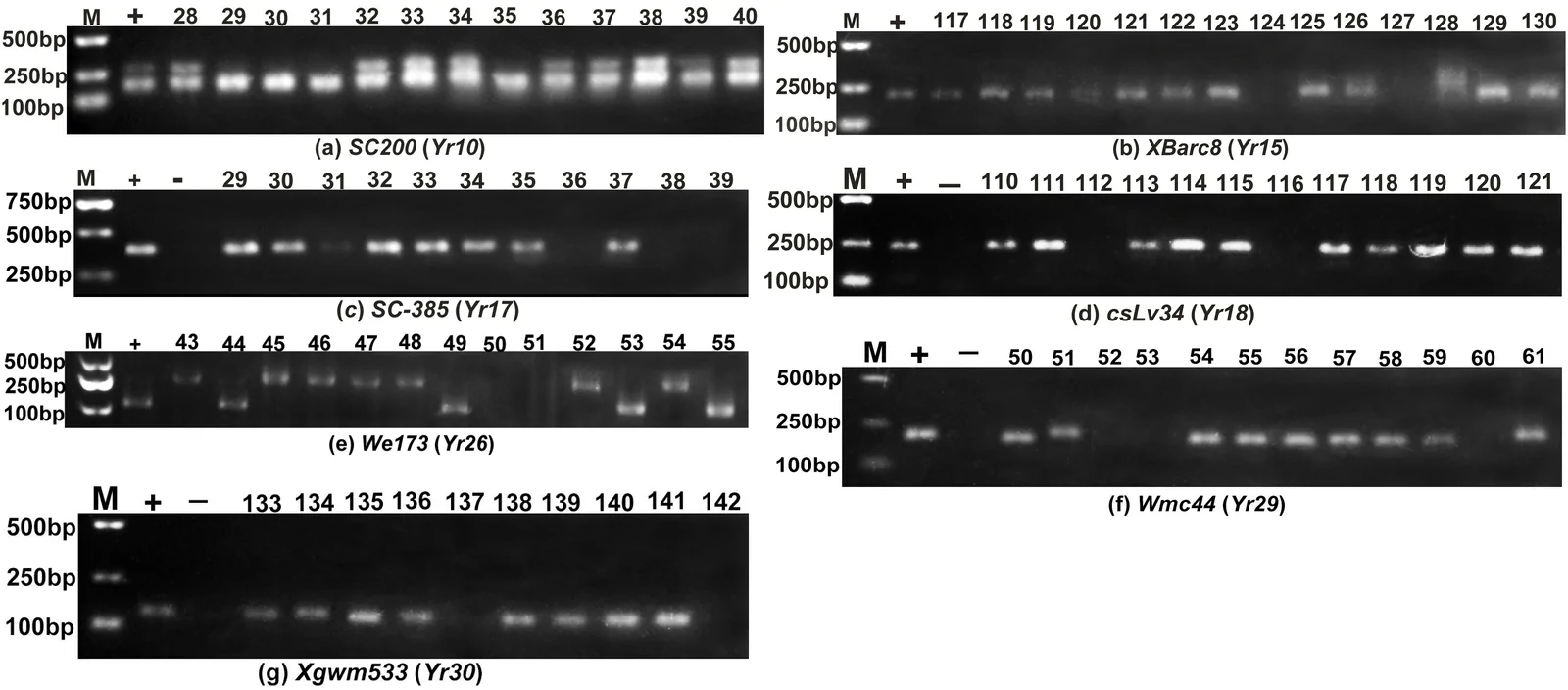
Molecular marker detection results for partial F_6_ families from five hybrid combinations. **(a)** *SC200* (*Yr10*); **(b)** *XBarc8* (*Yr15*); **(c)** *SC-385* (*Yr17*); **(d)** *csLv34* (*Yr18*); **(e)** *We173* (*Yr26*); **(f)** *Wmc44* (*Yr29*); **(g)** *Xgwn533* (*Yr30*). M: DNA molecular weight markers; +: positive controls were Chuanmai98 (CM98), Chuanyu34 (CY34), Xikemai11 (XKM11), and Longmai479 (LM479); -: negative control was Avocet S.

### 3.2 Evaluation of disease resistance at the adult plant stage

The parental families exhibited the following resistance profiles: LM479 showed moderate resistance (IT = 4–5, DS = 0–20%), while CM98, XKM11, and CY34 all displayed high resistance (IT = 1–3, DS = 0–20%) (**Figure 3**). Among the 164 F_6_ families, ten were immune (6.10%), 106 were highly resistant (64.63%), 23 were moderately resistant (14.02%), and 25 were susceptible (15.24%) (**Table 3**). The IT values for the five hybrid combinations were primarily concentrated between one and three. Taken together, the 164 screened materials exhibited a generally high level of APR (**Figures 4A, B**). Among the 32 F_6_ families from the cross LM479/CM98, no susceptible plants were observed. Of the 49 families from the cross LM479/XKM11, the numbers of immune, highly resistant, moderately resistant, and susceptible materials were 1, 38, 0, and 10, respectively. In terms of DS, DS values for the 164 families were predominantly concentrated between 0% and 20%, with the highest number of families in each of the five hybrid combinations falling within this range.

**Figure 3:**
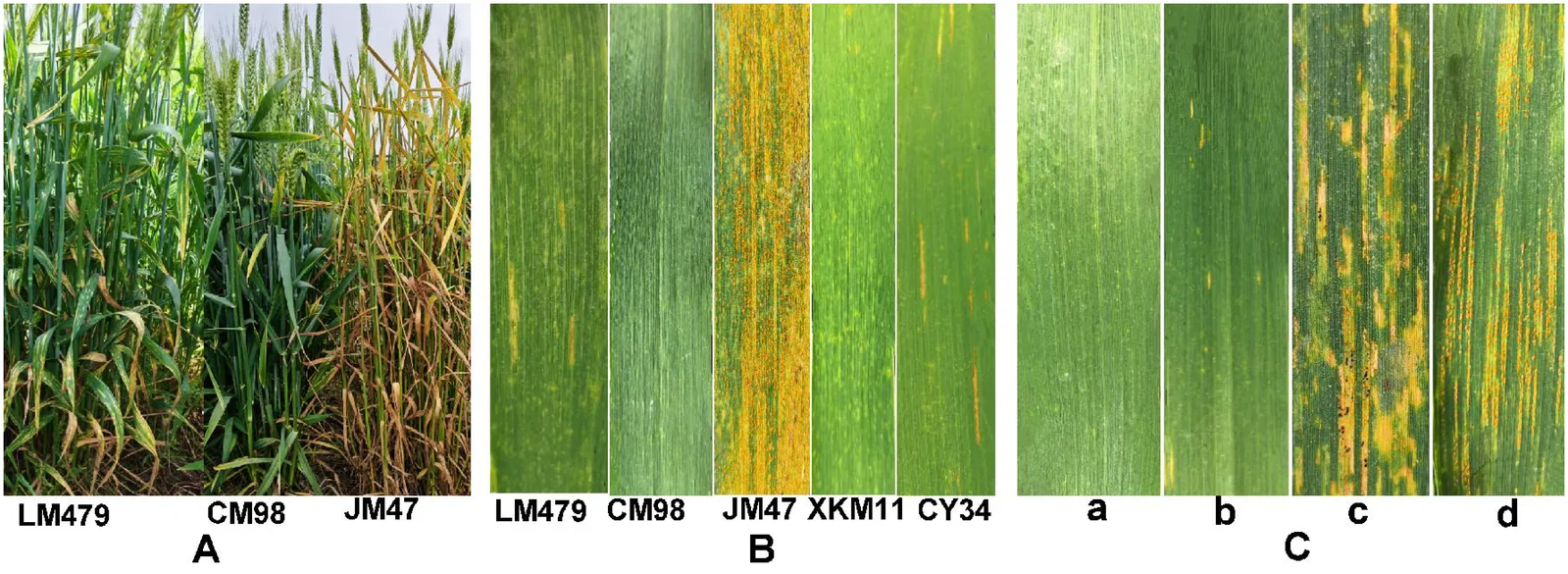
**(A)** Parents Longmai479 (LM479) and Chuangmai98 (CM98), and the control Jingmai47 (JM47); **(B)** Adult-plant leaf response types of the parents and the susceptible control; **(C)** Stripe rust resistance performance at the adult-plant stage in selected F_6_ families.

**Table 3:** Distribution of stripe rust resistance in 164 families of five hybrid combinations at the adult-plant stage (APR).

Cross combination	Immune (IT = 0)	Resistant (IT = 1-3)	Intermediate (IT = 4-6)	Susceptible (IT=7-9)	Total
LM479/50212-2	5	7	10	5	27
LM479/XKM11	1	38	0	10	49
LM479/CM98	2	26	4	0	32
LM479/CY34	1	17	1	5	24
LM479/50212-12	1	18	8	5	32

LM479, Longmai479; CM98, Chuanmai98; XKM, Xikemai11; CY34, Chuanyu34.

**Figure 4:**
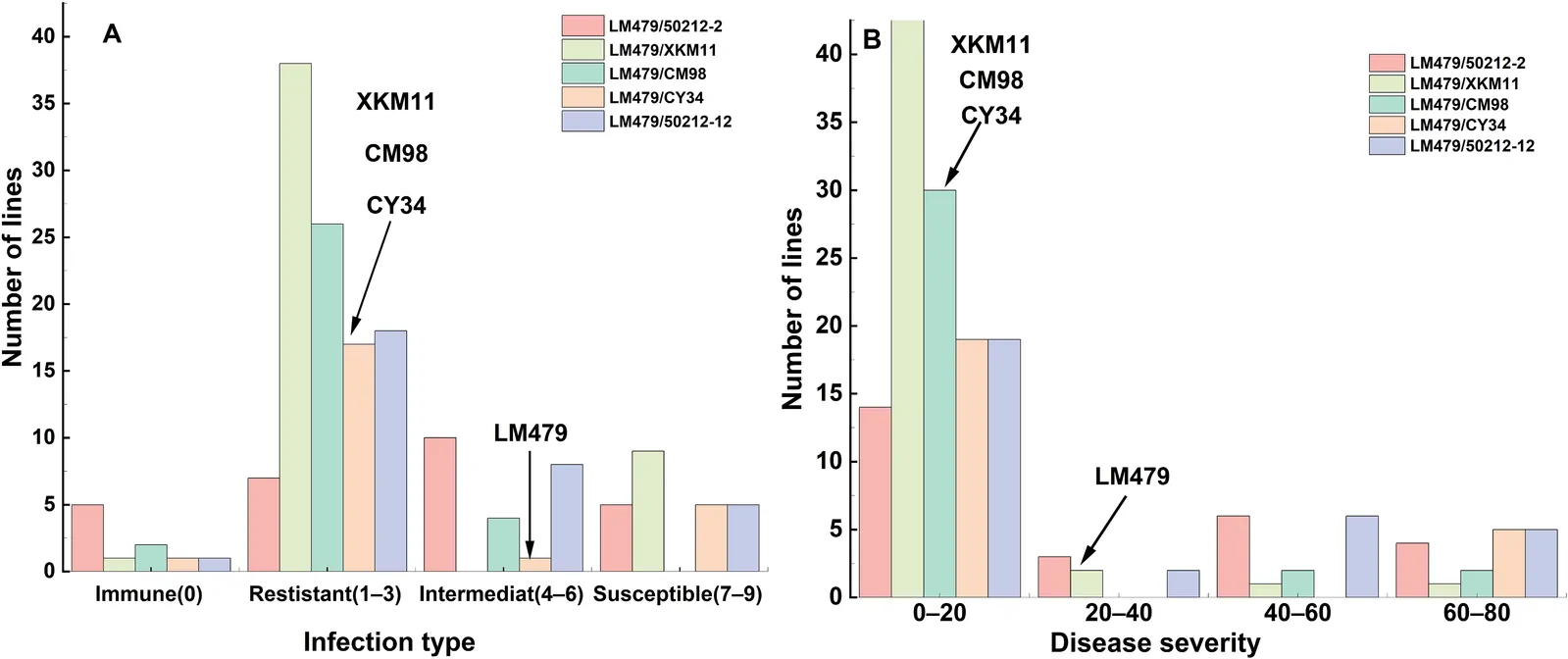
Frequency distributions of **(A)** infection type (IT) and **(B)** disease severity (DS) for adult- plant resistance (APR) in 164 F_6_ families derived from five hybrid combinations.

### 3.3 Effects of disease-resistance gene pyramiding on disease resistance

To elucidate the effect of pyramided *Yr* genes on APR, the 164 families were grouped according to the number of *Yr* genes carried, and differences in IT and DS among groups were compared (**Figures 5A, B**). Regarding IT, no significant differences were observed among families carrying different gene combinations. In terms of DS, no significant difference was found between families lacking *Yr* genes and those carrying only one *Yr* gene; however, significant differences were observed when comparing families lacking *Yr* genes with those carrying two, three, or four *Yr* genes, indicating that multi-gene pyramiding can effectively reduce the severity of stripe rust in the field.

**Figure 5:**
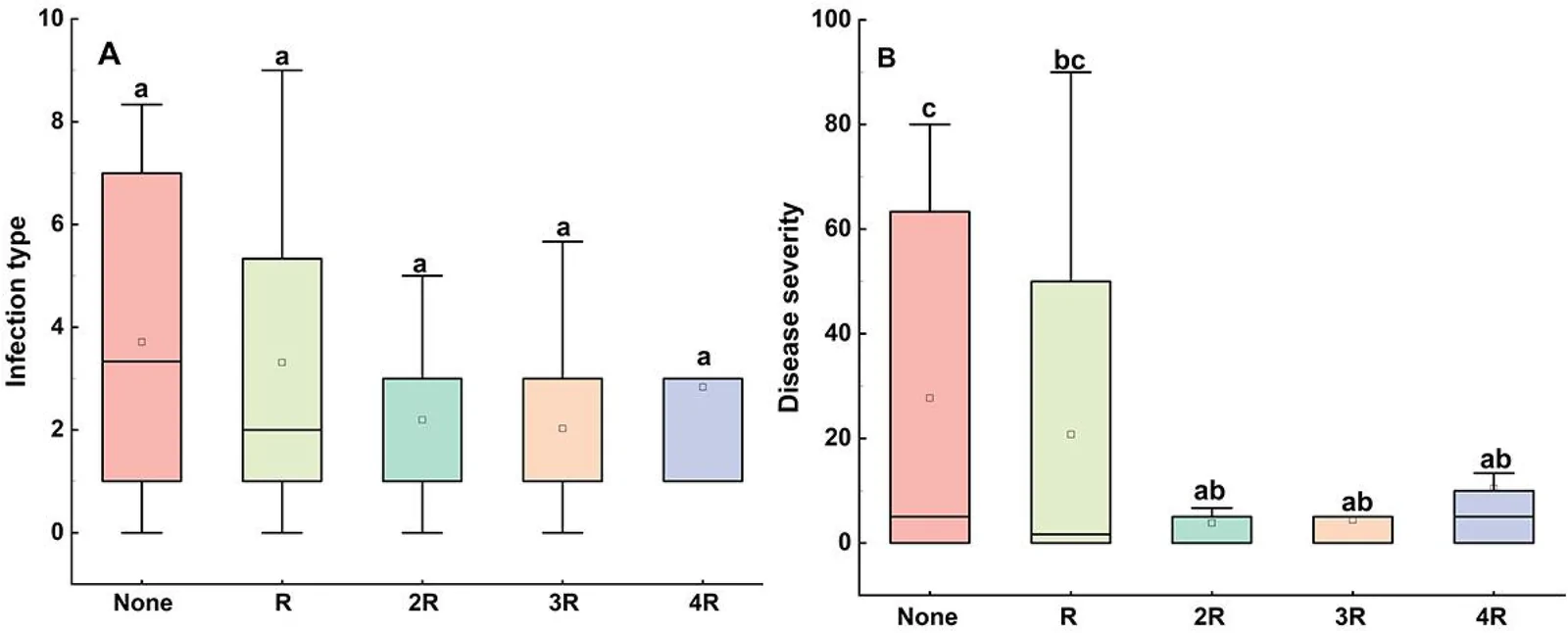
Comparison of infection types among 164 F_6_ families containing different *Yr* gene pyramids: **(A)** infection type (IT); **(B)** disease severity (DS). One-way analysis of variance (ANOVA) was applied for statistical comparisons. Different lowercase letters above the bars denote significant differences among groups at P < 0.05, while the same letters indicate non-significant differences.

### 3.4 Agronomic trait evaluation

This study investigated the agronomic traits of 164 F_6_ families derived from five hybrid combinations, including PH, NT, SL, SN, TGW, and GNPS. The family frequency distributions for each trait are presented below (**Figures 6A–F**). The results are as follows. PH: The values were predominantly concentrated in the 80–100 cm range. For the LM479/50212-2, LM479/XKM11, and LM479/CY34 families, the highest proportion fell within the 80–90 cm range, with 15, 23, and 13 families, respectively. For the LM479/CM98 and LM479/50212–12 families, the highest proportion was observed in the 90–100 cm range, with 21 and 18 families, respectively. NT: All families were primarily distributed within the range of four to eight tillers. Across all five hybrid combinations, the largest number of families exhibited four to six fertile tillers, indicating a generally balanced tillering capacity across the population. SL of the families was predominantly distributed in the 9–13 cm range. The LM479/XKM11, M479/CM98, LM479/CY34, and LM479/50212–12 families showed the highest proportions in the 9–11 cm range, with 28, 22, 14, and 7 copies, respectively. No materials with SL ≥13 cm were observed in the first three crosses.

**Figure 6:**
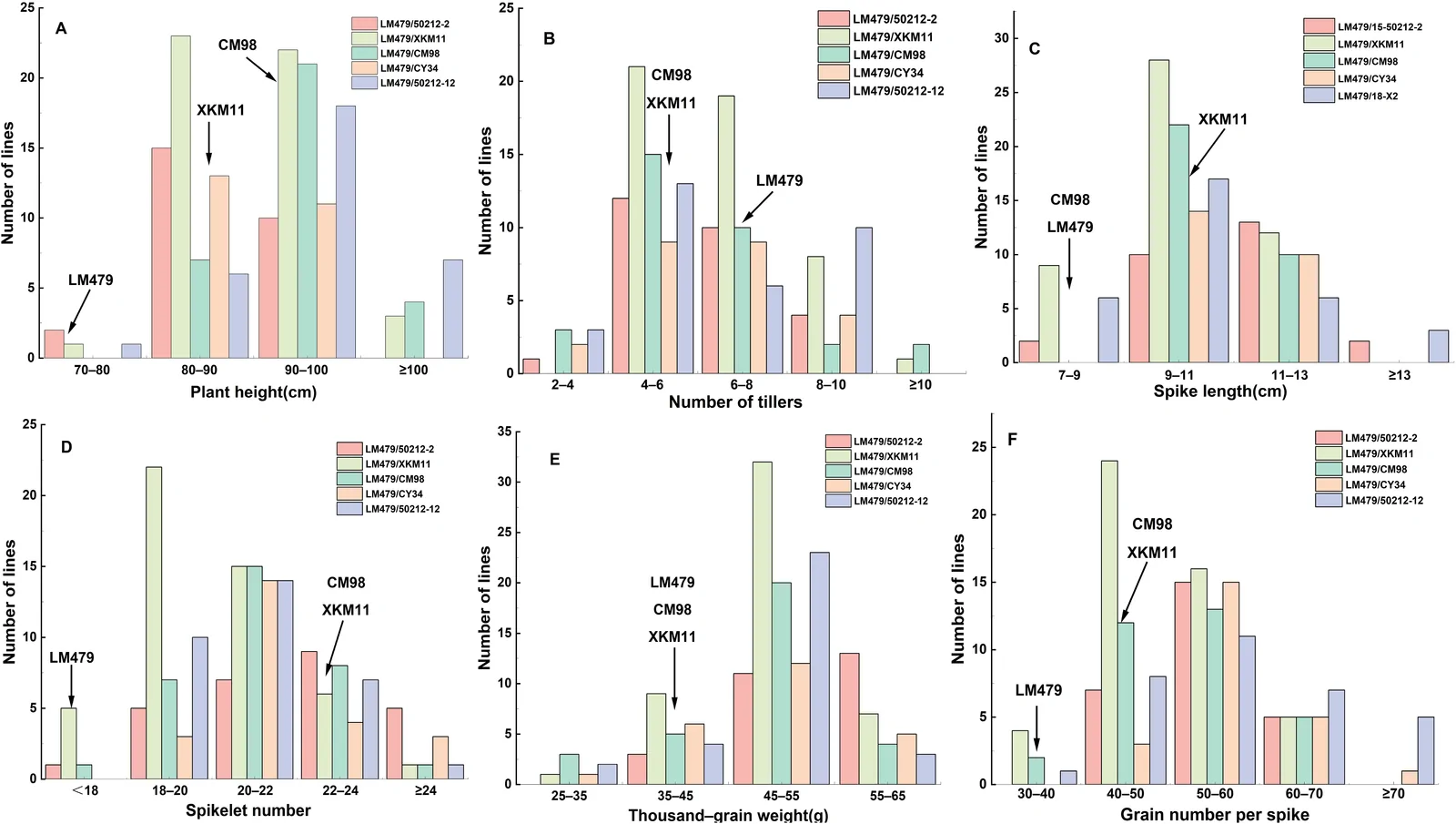
Distribution of agronomic traits across 164 F_6_ families derived from five hybridization combinations. **(A)** Plant height (PH); **(B)** number of tillers (NT); **(C)** spike length (SL); **(D)** spikelet number (SN); **(E)** thousand-grain weight (TGW); **(F)** grain number per spike (GNPS).

Number of fertile SN: The population had 18–24 fertile spikelets. The LM479/XKM11 family had the highest proportion in the 18–20 fertile spikelet range (22 copies), whereas the other four crosses had the largest number of families in the 20–22 fertile spikelet range. TGW: For all five hybrid combinations, the highest proportion of materials fell within the 45–55 g range, with 11, 32, 20, 12, and 23 copies for LM479/50212-2, LM479/XKM11, LM479/CM98, LM479/CY34, and LM479/50212-12, respectively. The TGW levels met the conventional standards for high-yielding wheat in the Sichuan wheat-growing region. GNPS: The LM479/XKM11 family exhibited the highest proportion in the 40–50 grain range (24 copies), whereas the other four crosses had the greatest number of families in the 50–60 grain range. Notably, no materials with more than 70 grains per spike were observed in LM479/50212-2, LM479/XKM11, or LM479/CM98. Overall, these families pyramiding stripe rust resistance genes displayed balanced agronomic traits, meeting the production and planting requirements of the plain and hilly wheat-growing regions in Sichuan.

### 3.5 Effects of disease-resistance gene pyramiding on agronomic traits

To clarify the impact of pyramiding *Yr* genes on core agronomic traits for stripe rust resistance, this study grouped 164 tested families by the number of *Yr* genes carried. Based on prior identification of APR, a systematic analysis of variance was conducted to evaluate the effects of different *Yr* gene pyramiding combinations on agronomic traits (**Figures 7A–F**). The analysis results indicated no significant differences among different *Yr* gene pyramiding combinations in PH, NT, SL, GNPS, TGW, and SN. These results indicate that although certain numerical differences exist in the agronomic traits among different combinations of pyramided *Yr* genes, the overall variation remains within a narrow range. Consequently, pyramiding stripe rust resistance genes did not significantly affect the major agronomic traits.

**Figure 7:**
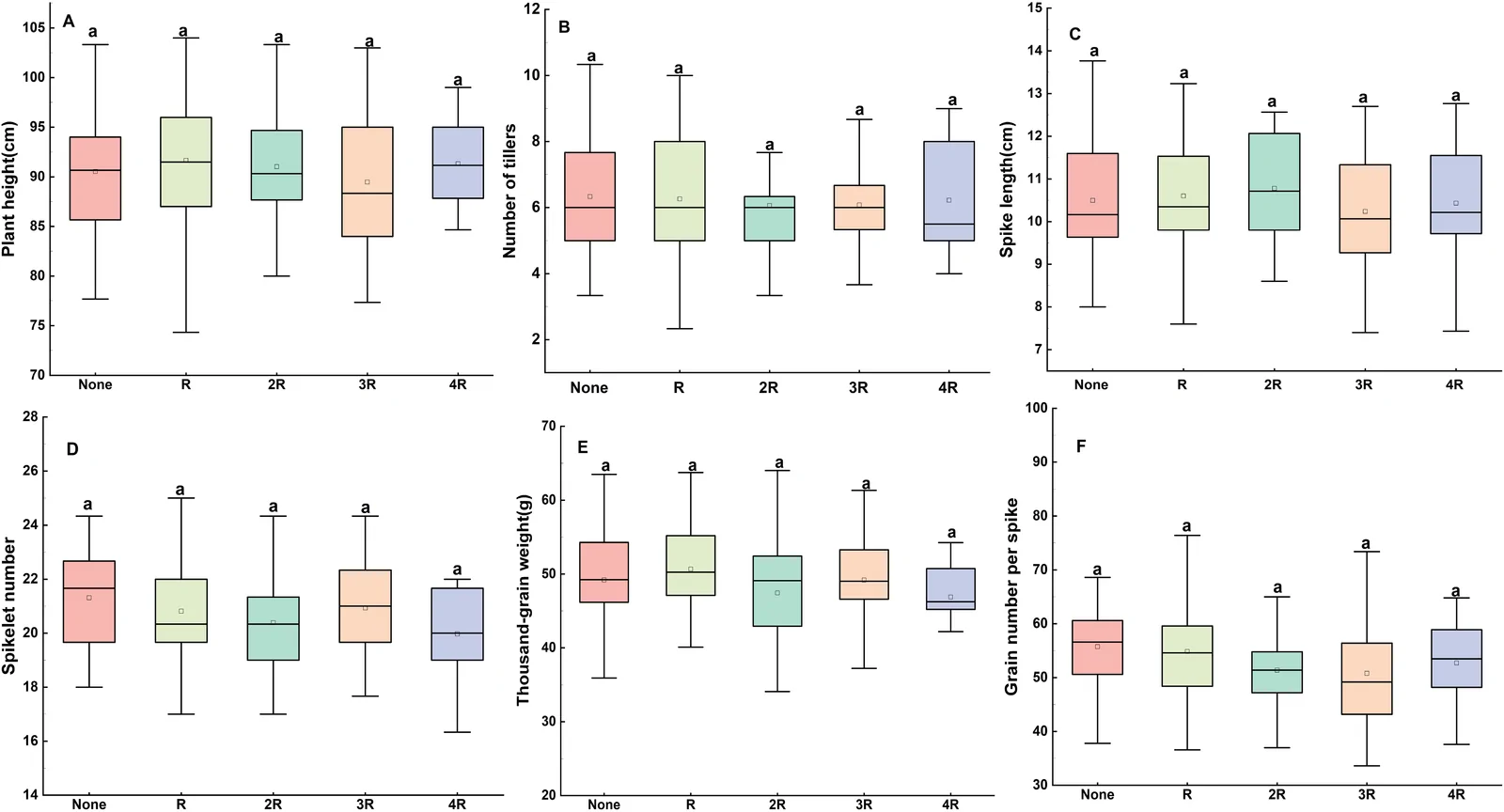
Comparison of agronomic traits among F_6_ families with different numbers of pyramided *Yr* genes. **(A)** Plant height (PH); **(B)** number of tillers (NT); **(C)** spike length (SL); **(D)** spikelet number (SN); **(E)** thousand-grain weight (TGW); **(F)** grain number per spike (GNPS). One-way analysis of variance (ANOVA) was applied for statistical comparisons. Different lowercase letters above the bars denote significant differences among groups at P < 0.05, while the same letters indicate non-significant differences.

## 4 Comprehensive selection of breeding materials

To screen for new wheat germplasm with disease resistance and superior agronomic traits, this study conducted a comprehensive selection of 164 F_6_ families based on common wheat breeding selection criteria: IT ≤ 3, DS ≤ 20%, PH between 75 and 95 cm, NT ≥ 4, SL ≥ 12 cm, SN ≥ 18, TGW ≥ 45 g, and GNPS ≥ 45. Ultimately, 18 elite DS families were identified (**Table 4**), which can be directly applied to subsequent wheat resistance pyramiding breeding. was as follows: two, six, six, three, and one family from LM479/50212-2, LM479/XKM11, LM479/CM98, LM479/CY34, and LM479/50212-12, respectively. Based on the number of *Yr* genes carried, the families were distributed as follows: two lacked any target genes (both from the LM479/50212–2 cross), two carried one, seven carried two, four carried three, and three carried four.

**Table 4:** The 18 selected F_6_ lines, their *Yr* gene composition, stripe rust resistance, and agronomic traits.

Line	Stripe rust resistance			Agronomic traits					
	Gene	IT	DS (%)	PH (cm)	NT	SL (cm)	SN	TGW (g)	GNPS
LM479/50212-2-19	—	1.00 ± 0.00	0.00 ± 0.00	85.00 ± 0.00	7.00 ± 1.00	12.40 ± 0.89	24.33 ± 1.76	46.77 ± 0.97	57.20
LM479/50212-2-25	—	1.00 ± 0.00	0.00 ± 0.00	83.67 ± 1.86	8.33 ± 0.88	12.10 ± 0.15	22.67 ± 1.20	51.43 ± 1.71	67.00
LM479/XKM11-33	*Yr10*+*Yr17*	1.00 ± 0.00	0.00 ± 0.00	94.67 ± 0.33	6.00 ± 1.53	12.33 ± 0.13	22.67 ± 0.33	50.73 ± 3.35	52.60
LM479/XKM11-41	*Yr10*+*Yr17*+*Yr29*+*Yr30*	1.00 ± 0.00	0.00 ± 0.00	90.67 ± 1.67	5.00 ± 0.58	12.27 ± 0.12	21.67 ± 0.33	45.87 ± 1.54	54.80
LM479/XKM11-65	*Yr10*+*Y17*	1.00 ± 0.00	0.00 ± 0.00	91.67 ± 3.38	6.33 ± 0.67	12.03 ± 0.32	22.67 ± 0.33	45.20 ± 0.85	49.20
LM479/XKM11-66	*Yr10*+*Y17*	1.00 ± 0.00	0.00 ± 0.00	88.33 ± 0.88	5.33 ± 0.33	12.57 ± 0.07	20.00 ± 0.58	56.47 ± 1.25	54.80
LM479/XKM11-70	*Yr17*+*Yr26*+*Yr29*	2.00 ± 0.00	0.00 ± 0.00	88.00 ± 1.73	7.33 ± 1.45	12.37 ± 0.86	20.67 ± 0.88	47.90 ± 1.46	68.60
LM479/XKM11-72	*Yr17*+*Yr30*	3.00 ± 0.00	5.00 ± 0.00	86.00 ± 0.58	6.00 ± 1.00	12.43 ± 0.12	19.00 ± 1.15	49.13 ± 1.75	53.40
LM479/CM98-86	*Yr10*+*Yr17*+*Yr29*	2.67 ± 0.33	3.33 ± 1.67	94.33 ± 0.67	5.33 ± 0.33	12.10 ± 0.06	20.33 ± 0.33	46.63 ± 0.73	48.00
LM479/CM98-87	*Yr10*+*Yr17*+*Yr26*+*Yr29*	3.00 ± 0.00	6.67 ± 1.67	94.67 ± 0.33	4.67 ± 0.33	12.77 ± 0.34	20.33 ± 0.33	45.23 ± 0.38	63.00
LM479/CM98-88	*Yr10*+*Yr17*+*Yr26*+*Yr29*	3.00 ± 0.00	5.00 ± 0.00	93.00 ± 1.53	5.00 ± 0.58	12.43 ± 0.27	21.67 ± 0.33	46.10 ± 1.18	63.80
LM479/CM98-93	*Yr10*+*Yr30*	3.00 ± 0.00	5.00 ± 0.00	92.33 ± 3.18	5.67 ± 0.67	12.03 ± 0.03	20.33 ± 0.67	56.20 ± 1.07	64.60
LM479/CM98-100	*Yr17*+*Yr26*	3.00 ± 0.00	5.00 ± 0.00	94.00 ± 1.15	7.67 ± 1.76	12.50 ± 0.68	25.33 ± 0.88	64.03 ± 4.52	60.40
LM479/CM98-102	*Yr10*+*Yr17*+*Yr30*	3.00 ± 0.00	5.00 ± 0.00	94.33 ± 0.88	7.00 ± 0.58	12.43 ± 0.18	25.33 ± 0.88	53.30 ± 2.17	55.00
LM479/CY34-116	*Yr30*	1.33 ± 0.33	0.00 ± 0.00	84.00 ± 1.00	4.00 ± 0.67	12.43 ± 0.17	19.33 ± 0.88	58.77 ± 1.05	57.80
LM479/CY34-121	*Yr15*+*Yr18*+*Yr30*	1.00 ± 0.00	0.00 ± 0.00	95.00 ± 1.73	6.33 ± 0.33	12.30 ± 0.10	22.67 ± 0.88	49.60 ± 2.54	53.40
LM479/CY34-122	*Yr15*+*Yr18*	1.00 ± 0.00	0.00 ± 0.00	82.67 ± 1.45	5.00 ± 0.58	12.10 ± 0.26	24.33 ± 0.88	45.27 ± 0.66	52.40
LM479/50212-12-163	*Yr30*	2.00 ± 0.00	0.00 ± 0.00	90.67 ± 1.86	6.67 ± 0.88	13.23 ± 0.57	20.67 ± 1.67	46.10 ± 0.82	48.80

IT, infection type; DS, disease severity; PH, plant height; NT, Number of tillers; SL, spike length; SN, spikelet number; TGW, thousand-grain weight; GNPS, Grain number per spike; LM479, Longmai479; CM98, Chuanmai98; XKM, Xikemai11; CY34, Chuanyu34.

## 5 Discussion

### 5.1 Resistance to wheat stripe rust

Wheat is the most widely distributed staple crop globally, providing calories and protein for approximately 30% of the world’s population (He et al., 2018). Stripe rust can cause wheat yield losses exceeding 40% or even total crop failure (Chen, 2005). In recent years, with the emergence of new virulent races such as CYR32, CYR33, and CYR34, the resistance of most major cultivated varieties in China has gradually declined. This phenomenon is directly related to the rapid variation in Pst physiological races and the complex composition of their populations, posing a direct threat to food security in major production areas (Liu et al., 2017; Gao et al., 2023; Wang et al., 2019).

Currently, many varieties carrying *Yr10* and *Yr26*, such as the Chuanmai series and the 92R series, have lost field resistance due to the emergence of new physiological races CYR33 and CYR34 (Wang et al., 2019; Chen et al., 2009; Bai et al., 2018). *Yr17* has also lost effective resistance to CYR32. Only a few *Yr* genes, such as *Yr5* and *Yr15*, confer ASR to all reported physiological races of wheat stripe rust in China (Hu et al., 2022; Huang et al., 2020; Fang et al., 2011). Resistance identification and molecular detection of 153 domestic and international wheat accessions by Zhou et al. (2024) revealed that the resistance sources in domestically bred cultivars were still predominantly *Yr9*, *Yr10*, and *Yr26*. Among these, although the ASR gene *Yr10* has lost resistance to the highly pathogenic race CYR34, it maintains a high level of resistance against the currently widespread races CYR32 and CYR33 (Liu et al., 2017). In contrast, resistance conferred by *Yr26* has been completely overcome by the prevalent race CYR34; therefore, this gene should not be used alone in breeding, and multiple resistance genes need to be pyramided to develop cultivars with durable resistance. In this study, the genes *Yr26* and *Yr10* were detected in the families of two hybrid combinations, LM479/XKM11 and LM479/CM98; however, the detection rate of *Yr26* was low (38.3%), and these two genes were pyramided with other *Yr* genes.

### 5.2 Molecular marker-assisted breeding and molecular markers

Compared with conventional phenotypic evaluation, MAS can markedly improve the efficiency and accuracy of pyramiding multiple resistance genes in breeding, offering a reliable approach to enhancing disease resistance in wheat. To address the production needs in the Sichuan wheat-growing region, where stripe rust is highly prevalent, Wang et al. (2023) used functional gene markers to pyramid three stripe rust resistance gene families, lines *Yr28*, *Yr36*, and *Yr18*, into Shumai969 and Shumai830, successfully obtaining families resistant to the disease throughout all growth stages. Zeng et al. (2005) also efficiently screened for innovative wheat germplasm with multiple pyramided resistance loci through multi-round hybridization and complex crosses combined with marker selection. In this study, a strategy combining conventional breeding with MAS was employed to select families containing target gene pyramids from hybrid progeny, ultimately obtaining 18 families with good DS, superior agronomic traits, and high yield potential. Among these, two families contained no *Yr* genes, two contained one, seven contained two, four contained three, and three contained four.

*Yr29* confers APR to stripe rust, leaf rust, stem rust, and powdery mildew (Zhang et al., 2019). The single-gene resistance conferred by *Yr18* and *Yr30* is weak, with high susceptibility at the seedling stage; however, their pyramiding can enhance DS through additive effects (Liu et al., 2024). *Yr17/Lr37* does not affect TGW or GNPS and can mitigate disease-induced losses, making it suitable for breeding programs aimed at high yield and DS. Ideal molecular markers should exhibit high polymorphism and maintain the closest possible linkage distance to the target gene. In this study, some families already pyramided with multiple *Yr* genes exhibited high or moderate susceptibility to stripe rust in the field, failing to achieve near-immunity levels. This discrepancy may be attributed to the genetic distance between markers and target genes; for instance, *WMC44* is located 5–15 cM from *Yr29* (Rosewarne et al., 2006), and *WMS533* is 2.0 cM from *Yr30* (Hayden et al., 2004). The remaining physical distance between linked markers and the target stripe rust resistance genes may lead to false-positive results in MAS for individual plants. Furthermore, among the 18 screened families, two did not test positive for *Yr* genes yet exhibited excellent family resistance. This suggests the potential presence of novel resistance genes that have not yet been detected or characterized, or the existence of unknown genes in the parental families. This study screened 164 families and identified 72 families carrying *Yr30.*

### 5.3 Gene pyramiding breeding

Pyramiding resistance genes effective throughout the entire growth period and at the adult plant stage to construct a synergistic defense system is a key strategy for breeding wheat with durable resistance to stripe rust. Liu et al. (2024) confirmed that pyramiding the pleiotropic gene clusters *Lr27/Yr30/Sr2/Sb3* and *Lr34/Yr18/Pm38/Sr57* can significantly enhance broad-spectrum resistance at the APR. Zhou et al. (2024) found that while resistance in major domestic cultivars relies mainly on a few genes (*Yr9*, *Yr10*, *Yr26*), CIMMYT families have achieved durable resistance through pyramiding one or two seedling non-immune genes with two or three APR genes—a practice that can serve as a reference for domestic breeding. Kokhmetova et al. (2021) also demonstrated that Kazakhstani variety combinations such as *Yr5*+*Yr17*+*Yr18* confer moderate to high levels of resistance. In this study, although *Yr10* and *Yr26* individually failed against the new CYR34 race, their pyramiding with effective genes such as *Yr17*, *Yr18*, *Yr29*, and *Yr30* still significantly improved overall resistance without affecting key agronomic traits, including PH, NT, SL, SN, TGW, and GNPS, providing a basis for multi-gene pyramiding breeding using partially ineffective resistance sources in the southwestern wheat region of China. In this study, regarding DS, no significant difference was observed between the non-carrier and single-gene-carrier groups; however, non-carriers differed significantly from families carrying two, three, or four genes. These findings confirm that pyramiding multiple genes can effectively mitigate the risk of rapid breakdown associated with single-gene resistance.

### 5.4 Limitations of this study

This study has several limitations. First, physiological races or pathotypes of Pst naturally occurring in the field were not isolated and identified during the experimental period. Because the population structure of this pathogen shows spatiotemporal dynamics, dominant races may different years and regions, which in turn may reduce the accuracy and representativeness of the resistance evaluation. Second, under ideal conditions without selection and with unbiased segregation, the homozygosity rate at DNA loci reaches 93.75% in the F_5_ generation and 96.875% in the F_6_ generation. Since the tested materials have not attained complete homozygosity, genetic segregation may still occur in the F_6_ generation. In addition, agronomic traits were assessed using only three plants per plot, giving a relatively small sample size. High within-family consistency was observed after selection in the F_5_ generation, and sampling error was reduced by strictly selecting plants from the middle rows while excluding border plants; nevertheless, a larger sample size would further improve the accuracy and reliability of trait evaluation. Future studies should strengthen the monitoring of strains prevalent in the field. Moreover, when DS phenotypes are identified, intermediate transitional types between moderate resistance (MR) and moderate susceptibility (MS) are often difficult to distinguish clearly. This ambiguity is largely attributed to the strong influence of environmental conditions, such as temperature, humidity, and inoculation density, on stripe rust infection.

## 6 Conclusions

Using LM479 as the female parent, five hybrid combinations were configured with Sichuan wheat varieties to construct a genetic population comprising 164 families. The integration of conventional breeding with MAS facilitated the pyramiding of multiple resistance genes into the F_6_ generation, resulting in 18 new wheat families with durable stripe rust resistance and desirable agronomic performance. Eighteen family lines were selected, of which seven carried the *Yr30* gene. The four hybrid combinations LM479/XKM11, LM479/CM98, LM479/CY34, and LM479/50212–12 accounted for two, two, two, and one line, respectively.

## Data Availability

The original contributions presented in the study are included in the article/supplementary material. Further inquiries can be directed to the corresponding author.
